# Calcinosis Cutis and Calciphylaxis in Autoimmune Connective Tissue Diseases

**DOI:** 10.3390/vaccines11050898

**Published:** 2023-04-25

**Authors:** Ilaria Mormile, Francesca Mosella, Piergiorgio Turco, Filomena Napolitano, Amato de Paulis, Francesca Wanda Rossi

**Affiliations:** 1 Department of Translational Medical Sciences, University of Naples Federico II, 80131 Naples, Italy; 2 Department of Plastic and Reconstructive Surgery, University of Naples Federico II, 80131 Naples, Italy; francescamosella1@gmail.com (F.M.); drpiergiorgioturco@gmail.com (P.T.); 3 Center for Basic and Clinical Immunology Research (CISI), University of Naples Federico II, 80131 Naples, Italy; 4 WAO Center of Excellence, 80131 Naples, Italy

**Keywords:** autoimmune diseases, comorbidity, calcinosis cutis, rheumatoid arthritis, connective tissue diseases

## Abstract

Calcinosis represents a severe complication of several autoimmune disorders. Soft-tissue calcifications have been classified into five major types: dystrophic, metastatic, idiopathic, iatrogenic, and calciphylaxis. Autoimmune diseases are usually associated with dystrophic calcifications, including calcinosis cutis, occurring in damaged or devitalized tissues in the presence of normal serum levels of calcium and phosphate. In particular, calcinosis cutis has been described in dermatomyositis, polymyositis, juvenile dermatomyositis, systemic sclerosis, systemic lupus erythematosus, primary Sjögren’s syndrome, overlap syndrome, mixed connective tissue disease, and rheumatoid arthritis. Calciphylaxis, a severe and life-threatening syndrome presenting with vascular calcifications and thrombosis, has also been associated with some autoimmune conditions. Due to the potentially disabling character of calcinosis cutis and calciphylaxis, physicians’ awareness about the clinical presentation and management of these diseases should be increased to select the most appropriate treatment option and avoid long-term complications. In this review, we aim to analyze the clinical features of calcinosis cutis and calciphylaxis associated with autoimmune diseases, and the main treatment strategies evaluated up to now for treating this potentially disabling disease.

## 1. Introduction

Calcinosis represents a severe complication of several autoimmune disorders. The accumulation of calcinotic materials induces the formation of hard nodules, which may lead secondarily to skin ulcerations, muscle atrophy, and joint contractures. Calcinosis cutis may be complicated by recurrent episodes of local inflammation and infections causing pain and functional disability, considerably affecting patients’ quality of life [1,2,3]. Soft-tissue calcifications have been classified into five major types: dystrophic, metastatic, idiopathic, tumoral, and calciphylaxis [2,4]. Autoimmune diseases are usually associated with dystrophic calcifications, including calcinosis cutis, occurring in presumably damaged or devitalized tissues in the presence of normal serum levels of calcium and phosphate [2]. Calciphylaxis, also known as calcifying uremic arteriolopathy, has also been observed [5,6,7]. Calciphylaxis is a severe and life-threatening syndrome presenting with vascular calcifications and thrombosis. It is characterized by the occlusion of microvessels in the subcutaneous adipose tissue and dermis, which causes intensely painful necrotic skin ulcers. The etymology of the word “calciphylaxis” is derived from “*calci*”, a Latin word semantically related to the process of calcification, and “*phylaxis*”, a Greek word meaning protection. The term means protection by calcification and was first used by Dr. Seyle [8,9] in 1961 to describe the formation of a calcium shell on rat skin [10]. Later, calcifying skin lesions developing in humans with renal failure were named calciphylaxis because of their similarities to calcified rat skin [11]. However, rather than a protective response, calciphylaxis in humans appears to be the skin equivalent of a myocardial infarction [11]. Calciphylaxis is rare, but it has a very high mortality rate due to the severe pain and propensity for infections, which lifts its annual mortality from 40% to 80% [12].

The disorder typically occurs in patients with end-stage renal disease (ESRD) treated with dialysis, a population with a high prevalence of extra-skeletal calcifications. Although calciphylaxis is strongly related to decreased kidney function, it is not only a severe complication of advanced diseases. Indeed, it also occurs in patients with earlier stages of chronic kidney disease, acute kidney injury, and prior receipt of a kidney transplant [13].

Regarding the association between calcinosis and autoimmune diseases, calcinosis cutis has been described in dermatomyositis (DM), polymyositis (PM), juvenile DM (JDM), systemic sclerosis (SSc), systemic lupus erythematosus (SLE), primary Sjögren’s syndrome (pSS), overlap syndrome, mixed connective tissue disease, and rheumatoid arthritis [14,15,16]. The autoimmune connective tissue disorders most frequently associated with calcinosis cutis are DM and SSc [17]. On the contrary, calciphylaxis is mainly observed as a consequence of renal failure, often associated with connective tissue disease, while non-uremic calciphylaxis is rare. However, the prevalence of this condition is often underestimated, resulting in delay in diagnosis and long-term complications.

In this review, we aim to analyze the clinical features of calcinosis cutis and calciphylaxis associated with autoimmune diseases and the main treatment strategies evaluated up to now for treating this potentially disabling disease.

## 2. Calcinosis Cutis and Systemic Sclerosis

SSc is a complex autoimmune connective tissue disease affecting the skin, gastrointestinal tract, and lungs, which carries potentially life-threatening complications [18,19]. Microvascular changes with endothelial cell dysfunction, followed by their transition into myofibroblasts, are central in the pathogenesis of the disease [20,21]. In addition, the development of progressive fibrosis and ischemia involving skin and other organs may lead to their irreversible failure [22]. Skin involvement is a prominent disease feature, including Raynaud’s phenomenon, digital ulcers, cutaneous sclerosis, telangiectasias, pruritus, dyspigmentation, and calcinosis cutis [18]. Calcinosis cutis may present in 18% up to 49% of SSc patients and is generally more prominent in patients with CREST syndrome (i.e., calcinosis, Raynaud’s phenomenon, esophageal dysmotility, sclerodactyly, and telangiectasia), and with anticentromere antibodies [1,23,24,25,26,27,28]. 

The calcinotic nodules are composed of calcium hydroxyapatite deposits, surrounded by inflammatory cells. They are usually localized at sites of recurrent microtrauma such as the forearms, elbows, or fingers [2,26].

When localized at the fingers, calcifications may be associated with ischemia and nerve pain [29]. Indeed, ischemia may play a role in the pathogenesis of calcinosis in SSc through the induction of increased production of oxidative stress products, as suggested by the association observed by many authors between calcinosis, digital ulcers/pitting scars [30,31,32], and late nailfold videocapillaroscopic pattern [32,33]. This hypothesis is supported by a pilot study showing reduced perfusion in the superficial skin layers involving calcinotic areas compared with non-calcinotic areas [34] and by another analysis conducted in 43 SSc patients describing a higher association between ulnar artery occlusion and radiograph-confirmed calcinosis [35]. Other mechanisms may include recurrent trauma and abnormalities in bone matrix proteins [28]. A prospective multicenter study of 568 consecutive SSc patients evaluated clinical features and associated complications of calcinosis cutis [36]. The authors reported that the most frequently involved sites were the hands (70%), particularly the thumbs (19%), representing a significant cause of functional dysfunction and a high burden of disability. The most common complications were tenderness and spontaneous extrusion of calcinosis through the skin, while infections were rare. They also confirmed a strong association between calcinosis and digital ischemia. A recent study by Muktabhant et al. [37] aimed to identify common clinical features between SSc patients with and without calcinosis cutis and reported that patients with calcinosis cutis were significantly older than those without calcinosis cutis (every one-year increase of age provided the OR of calcinosis cutis detection of 1.05; 95% CI, 1.01–1.09). Interestingly, no association was found with the SSc subset or serology. Patients with SSc and calcinosis compared to SSc patients without calcinosis may show decreased serum levels of the mineralization inhibitors fetuin-A [23] and inorganic pyrophosphate [38] and, conversely, elevated levels of the mineralization promoter osteoprotegerin [26], suggesting systemic alterations in the extracellular matrix and mineral metabolism associated with this complication. In addition, higher serum fibroblast growth factor-23 (FGF-23) levels have also been found in SSc female patients with calcinosis [39].

Clinical features of calcinosis cutis may vary greatly among patients with SSc. Calcinotic nodules may be visible and palpable or only palpable [40]. A calcinosis classification according to depth and size was initially proposed by Berger et al. [41]. The authors identified four grades of calcinosis. First grade lesions are not palpable and only radiological; second grade includes multiple small palpable calcinotic nodules; patients with third grade calcinosis show larger and more numerous palpable calcifications; finally fourth grade include large deposits involving wider areas often complicated by ulceration [41]. This classification has been more recently updated by Bartoli and collaborators [40] based on the results derived from both clinical and X-ray evidence. According to the authors, calcinotic lesions may be classified into four groups: visible calcinosis not requiring an X-ray (mousse); usually palpable and not usually visible (net); possibly visible and/or palpable (plate); may be visible and/or palpable (stone). The support of X-rays is fundamental in the last three subset. 

**Figure 1:**
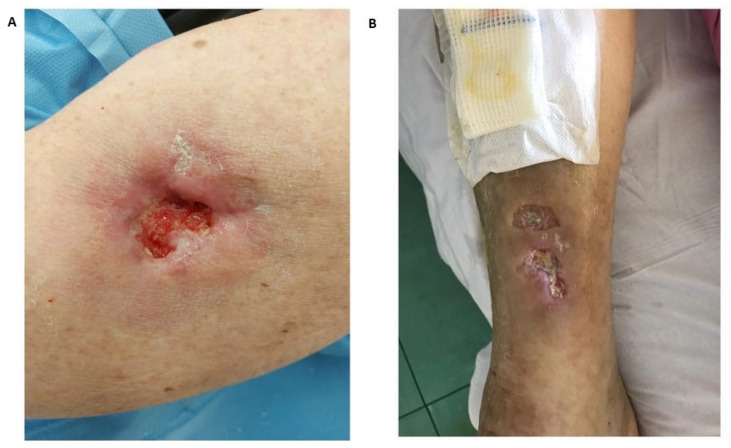
Cutaneous calcinosis in systemic sclerosis (SSc). Cutaneous calcinosis of the right superior limb complicated with ulceration in a female patient with morphea (**A**). Calcinotic masse of the right inferior limb in a male patient with SSc, complicated with two ulcerative lesions (**B**).

Inflammation is present in most patients’ skin surrounding the calcinotic area, and ulcers may develop in about half of the subjects (Figure 1) [40]. In these cases, a strict follow-up is required to lower the risk of complications such as infections and fistulation. 

Extensive diffuse calcifications (calcinosis cutis universalis) accumulating in the deep dermis, subcutaneous tissue, fascia, and muscles, are common in DM but only occasionally described in SSc [42,43,44]. Pseudoxanthoma elasticum-like calcification [45] and dystrophic calcinosis secondary to localized linear scleroderma have also been reported [46]. “Tumoral” or “pseudotumoral” calcinosis, consisting of multiple, large, and often symmetrical calcified masses, may occur in about 3% of SSc patients, more commonly in those with a severe vascular phenotype [47]. These masses are tough to treat and may be complicated by ulceration, infections, and nerve compression [47]. 

Calcinosis cutis in SSc patients have recently been linked with proton pump inhibitors (PPI) exposure [48]. The underlying pathogenetic mechanisms are unknown. However, it has been proposed that PPIs may induce tissue calcification promoting vascular endothelial cell damage, triggering pro-atherogenic pathways through the modulation of the chemokine secretory phenotype, altering endothelial lysosomal acidification, enzymatic activity, and proteostasis, causing endothelial senescence [49,50,51]. However, gastroesophageal reflux disease strongly impacts SSc patients’ morbidity [52,53], and further evaluations are awaited to guide the use of PPI, especially in patients with SSc at risk of progressive calcinosis.

Calcinosis in SSc is a major cause of morbidity, and many unmet needs in clinical practice remain unanswered. A better comprehension of the pathogenic mechanisms involved in this condition may help develop novel treatment approaches and identify reliable outcome measures and biomarkers to facilitate clinical trials [54].

## 3. Calcinosis Cutis and Poly-Dermatomyositis

PM and DM belong to the heterogeneous group of inflammatory myopathies [55]. They are both characterized by proximal limb and truncal muscle weakness and skeletal muscle chronic inflammation, generally in the absence of myalgia, impaired tendon reflexes, and sensory loss [56]. The skin, heart, gastrointestinal tract, and lungs may also be affected [56]. In addition, an increased risk of malignancies has been observed in DM patients [57]. 

Although showing some similarities, PM and DM differ in their clinical features, histopathology, response to treatment, and prognosis [56]. DM has several typical skin manifestations, usually affecting extensor surfaces and photo-exposed areas, including poikiloderma (i.e., a tetrad of hypopigmentation, hyperpigmentation, telangiectasias, and epidermal atrophy), nail fold telangiectasias, photosensitivity, pruritus, and calcinosis cutis [58]. Violaceous poikiloderma localized around the eyes is known as the “heliotrope sign”. Otherwise, when it occurs over the knuckles, elbows, and knees, it is called “Gottron’s sign” [58]. 

Unlike DM, in which the rash eases its recognition, PM mimics many other myopathies, remaining a diagnosis of exclusion [55]. Serum muscle enzyme concentrations, electromyography, and muscle biopsy confirm the clinical diagnosis of PM and DM. In some cases of DM, a skin biopsy can be helpful [55]. Autoantibodies against ribonucleoproteins involved in protein synthesis (anti-synthetase) or translational transport (anti-signal-recognition particle) may be found in about 20% of PM and DM patients [59]. Anti-Jo-1 accounts for 80% of all the anti-synthetases [59]. 

**Figure 2:**
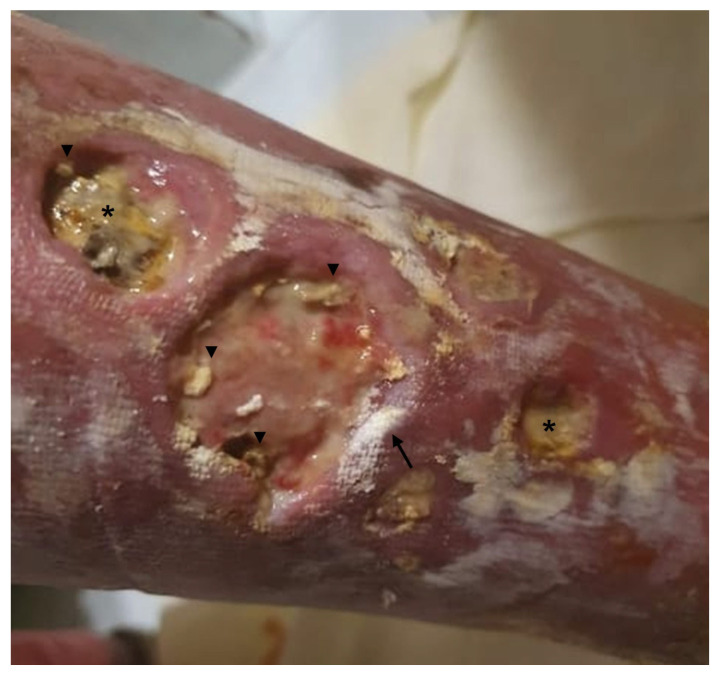
Cutaneous calcinosis in dermatomyositis (DM). Multiple and extensive calcinosis cutis (arrowheads) of the right inferior limb complicated with ulceration in a female patient with DM. Fibrinous exudate (asterisk) is present in the cavity of the wounds. The arrow indicates residues of antiseptic cream.

Calcinosis deposits in PM and DM may localize in the skin, deep fascia, or intramuscular connective tissue (Figure 2) [60]. 

Calcinosis cutis has been observed in both PM and DM, but it has been reported to be more common in DM than PM (DM 37% vs. PM 3.3%) [61] and in JDM (up to 70%) [62,63] than adult DM (up to 30%) [62,63,64]. On average, this complication shows an earlier presentation in JDM occurring 2–3 years after the onset of the disease. On the contrary, poly-dermatomyositis (PDM) appears about eight years after the diagnosis in adult patients [61]. Calcinosis universalis complicating severe DM may present as deep tumoral masses within the affected muscles or diffuse lacy reticular deposits within the myofascial planes, sometimes forming an extensive exoskeleton [2,65]. 

Cutaneous calcinosis may present unrelated to disease flares [66]. However, it is more common in DM patients with poor disease control, delay in diagnosis [67], a longer disease duration [68], dysphagia [69], and Gottron’s papules concomitance [61]. Laboratory predictors of the onset of calcinosis could be found in anti-NXP-2/MJ and PM/Scl [61,68]. In addition, the positivity for antibodies to NXP-2 is associated with calcinosis early onset and quick, widespread dissemination [61]. 

Pathogenetic mechanisms hypothesized for the development of calcinosis in PM/DM include inflammatory infiltrates in calcific deposits [70,71], local vascular ischemia [72], a dysregulation of mechanisms controlling the deposit/solubility of calcium and phosphate [73], and mitochondrial damage of muscle cells [74]. The expression of proinflammatory cytokines such as interleukin-1 (IL-1) and anti-tumor necrosis factor (TNF)-α, possibly induced by type I interferon (IFN I), has been described at the calcinosis site, suggesting macrophage activation [75]. In addition, the JAK-STAT (signal transducer and activator of transcription) pathway, especially acting through STAT3, may regulate mitochondrial calcium store release [76]. Moreover, STAT1 is overexpressed in the muscle tissue of DM patients [55,77]. Based on this evidence, JAK-inhibitor tofacitinib has been proposed as an attractive treatment choice in DM complicated by calcifications [78].

DM has been associated with the occurrence of malignancies in 13–42% of cases [79]. In this view, it is important to consider that calcinosis may be an unusual but possible paraneoplastic manifestation mainly associated with some hematological neoplasms [80]. In addition, two cases of malignancies (i.e., osteosarcoma and extranodal B-cell lymphoma) arising from an area of JDM-associated calcinosis have been described [81,82], showing that the [81] malignant transformation of benign heterotopic bone is extremely rare but possible. This scenario is further complicated as DM may also be part of a paraneoplastic syndrome, as some cases tend to improve when the primary neoplasm is treated. Because of the association between DM and malignancies, age-appropriate examinations and tests to screen for cancer are warranted in all patients with DM [55] since the prompt recognition of a paraneoplastic syndrome, including calcinosis, can lead to the detection of an otherwise clinically occult tumor at an early and highly treatable stage [83].

## 4. Calcinosis Cutis and Systemic Lupus Erythematosus

SLE is a chronic autoimmune disorder with a broad clinical spectrum involving multiple organs, including the skin, joints, kidneys, and central nervous system [84,85]. Ectopic calcinosis is a relatively common finding in patients with SLE; its prevalence is estimated at 40% [2,86,87,88]. Calcinosis is estimated to be more frequent in patients with lupus nephritis and vitamin D3 treatment [2,86,87,88]. The accumulation of calcinotic materials may develop at different sites, such as peripheral arteries, periarticular areas, and other soft tissues [87]. Conversely, cutaneous calcinosis in SLE is rare [89]; the dystrophic type is the most represented form, as in the other inflammatory disease, and it is generally circumscribed to limited areas of the skin [14,15,88]. Calcinosis universalis is extremely rare. It may present as a lace-like reticular or nodular type, and it is generally more common in female patients with a history of chronic active SLE [90]. Calcinotic lesions in SLE patients characteristically involve interphalangeal joints, forearms, elbows, buttocks, peri-auricular area, and tissue underneath cutaneous lupus lesions [91,92,93]. Facial calcinosis cutis has occasionally been described in SLE patients, even if it remains uncommon in all connective tissue disorders [94,95]. Calcinosis cutis is considered a late-stage complication, occurring on average 21.5 years after SLE onset [14]. The pathogenetic mechanisms involved in developing calcinosis cutis in SLE patients are not completely elucidated. Advanced glycation products and their receptors may be involved, as increased tissue expression has been observed in SSc and SLE patients with calcinosis cutis [88]. Other authors hypothesized that vascular alterations might cause ischemic changes leading to lipomembranous changes and calcification in the subcutaneous tissues [46,56]. Other research groups suggested that local tissue abnormalities, such as alterations in collagen or elastin may contribute to precipitating ectopic calcification, and phosphate bound to denatured proteins of necrotic cells at the site of trauma or inflammation may serve as a nidus for calcium deposition [96,97].

Understanding the underlying pathogenetic mechanisms involved in SLE-associated calcinosis cutis may help develop tailored treatment options. Surgical excision is currently the most common and effective choice [98]. Other strategies include topical and intralesional steroids, topical and intralesional sodium thiosulfate, colchicine, and calcium channel blockers [99].

## 5. Calcinosis Cutis and Other Autoimmune Conditions

Calcinosis cutis has been occasionally reported in several other autoimmune conditions such as pSS, RA, psoriatic arthritis, and autoinflammatory diseases.

pSS is a systemic autoimmune disease characterized by lymphocytic infiltration of the secretory glands, leading to mucocutaneous dryness [100] and extra-glandular manifestations, including musculoskeletal [101], pulmonary [102], renal [103], hematological [104], and neurological involvement [105]. Calcinosis cutis associated with pSS has occasionally been described. To date, there are four case reports of calcinosis cutis limited to fingertips [106], hands [107], and forearms [108]. In addition, two cases of massive calcinosis have been reported [109,110].

**Figure 3:**
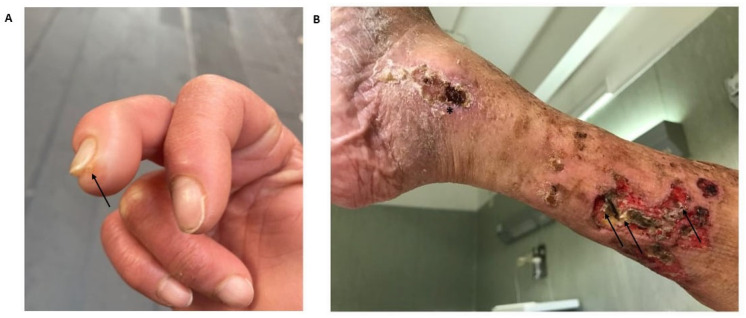
Cutaneous calcinosis in rheumatoid arthritis (RA). Calcinosis cutis in the soft tissue at the tip of the finger (arrow) in a female patient with RA (**A**). Multiple calcinotic lesions of the left leg (arrows) complicated with necrotizing ulcers in a female patient with RA. The asterisk indicates a concomitant chronic venous ulcer (**B**).

RA is a chronic autoimmune disease characterized by a progressive symmetric inflammation of the joints resulting in bone erosion, cartilage destruction, progressive loss of function, and joint deformity [111], mediated by both adaptative [112] and innate immunity [113,114,115]. Calcinosis cutis associated with RA is extremely rare, and few cases have been reported to date [14,16,116]. The calcinotic lesions may be observed in the extremities (Figure 3A), including the buttock, legs (Figure 3B), and elbow joint. 

Patients with psoriasis present a pathological bone remodeling induced by various cytokines, molecules, and cellular interactions [117]. Calcifications in patients with psoriasis and psoriatic arthritis are possible and mainly described in the coronary arteries and epicardial adipose tissue [118,119]. A few cases of calcinosis and cutaneous psoriasis have been previously described [120]. Calcinosis cutis in psoriatic arthritis is extremely rare. Azami et al. [121] reported a single case of a patient with psoriatic arthritis presenting with calcified masses with lobulated margins localized around the large joints mimicking malignant tumoral lesions (i.e., tumoral calcinosis). Another Italian research group reported a case of periarticular calcifications in the soft tissues at distal interphalangeal joints in a patient with psoriatic arthritis [122].

Autoinflammatory diseases are rare conditions caused by innate immunity abnormalities, which include familial Mediterranean fever, cryopyrin-associated periodic fever syndrome (CAPS) or NLRP3-associated autoinflammatory disease (NRLP3-AID), mevalonate kinase deficiency (MKD) and TNFRSF1A-receptor associated periodic fever syndrome (TRAPS), and periodic fever, aphthous stomatitis, pharyngitis, and cervical adenitis (PFAPA) syndrome [123]. The other conditions often included in this group since they have many similarities with hereditary autoinflammatory diseases are Still’s disease [124] and Schnitzler’s syndrome [125]. Calcinosis is generally uncommon in these patients. A single case of calcinosis cutis has been associated with adult-onset Still’s disease [126], and tumoral calcinosis of the gluteal region was reported in a female adolescent with juvenile polyarthritis [127], which shares the main symptoms (i.e., fever, arthritis, and evanescent salmon-colored skin rash) with Still’s disease. 

Table 1 summarizes epidemiological and clinical features of calcinosis cutis associated with autoimmune diseases.

In conclusion, despite their rarity in these conditions, calcinosis cutis is a remarkable cause of morbidity and functional impairment; therefore, it should always be suspected in patients with autoimmune diseases, especially when encountering non-healing extremity ulcers [16].

## 6. Calciphylaxis and Autoimmune Diseases

Calciphylaxis has been described in some autoimmune diseases such as antiphospholipid syndrome [5] and its life-threatening variant, catastrophic antiphospholipid syndrome (CAPS) [6]. Antiphospholipid syndrome is a complex syndrome characterized by the occurrence of venous and/or arterial thrombosis, pregnancy morbidity, and persistent antiphospholipid antibodies [132]. In this condition, about half of patients experience associated skin disorders [133], while renal involvement is generally considered less common and may range from 10–40% according to different cases [134]. The development of skin necrosis and dystrophic calcification secondary to renal dysfunction has been reported [5], bringing attention to calciphylaxis as a possible cause of ecchymotic-appearing skin lesions in antiphospholipid syndrome patients. Although the association between calciphylaxis and end-stage kidney disease is well-known, a case of non-uremic calciphylaxis associated with SLE and antiphospholipid syndrome has recently been reported [135]. The authors hypothesized that a deficiency of matrix Gla protein (MGP), which is a potent inhibitor of vascular calcification depending on vitamin K [136], induced by the use of glucocorticoids and warfarin, can be involved in the development of calciphylaxis [135]. 

Non-uremic calciphylaxis has also been reported in patients with other autoimmune conditions such as RA [137,138], SLE [139,140,141], and the concomitance of multiple rheumatologic disease (i.e., SLE, secondary Sjogren’s syndrome, and myasthenia gravis) [142]. Some proposed or speculated additional mechanisms leading to the development of non-uremic calciphylaxis are D hypovitaminosis and secondary hyperparathyroidism, often associated with mild renal disjunction occurring in patients with autoimmune diseases, and the mild hypercoagulable state induced by the development of anti-protein S antibodies in patients with RA [142].

## 7. Therapeutic Approaches for Calcinosis Cutis and Calciphylaxis in Autoimmune Conditions

Calcinosis cutis spontaneous resolution occurs very rarely. Specific treatments for calcinosis associated with autoimmune disease are currently lacking, and surgical management is often the best option [143]. Definite benefits with symptomatic relief and no recurrence in follow-up years have been described after surgical excision of the lesions [89,98,144]. Surgical treatments are especially indicated in cases of recurrent infections, ulcerations, painful masses, functional impairment, and cosmetic purposes [71,144]. However, surgical excision is limited to circumscribed lesions; hence it is not the first treatment consideration in extensive dystrophic calcinosis [15,145].

Although many different drugs have been used to treat rheumatic disease-associated calcinosis, such as colchicine, probenecid, bisphosphonates, diltiazem, minocycline, aluminum hydroxide, salicylate, and carbon dioxide laser therapies, none of them has convincingly prevented or reduced it [2,146]. For example, some case reports and case series mainly conducted in patients with calcinosis and JDM have shown contrasting results with the use of bisphosphonates [14,147,148,149]. The rationale for administering these drugs in these types of patients is the inhibition of macrophage proinflammatory cytokine production and reducing calcium turnover [4]. The human monoclonal antibody targeting the key bone resorption mediator RANKL, denosumab, has been reported to be effective in a patient with multiple sclerosis and metastatic calcinosis cutis due to refractory hypercalcaemia [150], but its efficacy remains to be explored in patients with calcinosis secondary to connective tissue disease Other drugs, such as warfarin, are currently not recommended [143].

Sodium thiosulfate intralesional injection seems a promising therapeutic option, especially for SSc-associated calcinosis cutis, leading to complete lesion resolution with scarce adverse effects [131,151]. In addition, the intravenous administration of sodium thiosulfate, used in association with wound care, surgical debridement, antibiotics, and cinacalcet, has also been shown to significatively improve the local outcomes and survival in a case series of patients with uremic calciphylaxis [152]. These results align with other case series by Baldwin et al. [153], reporting full remission of skin lesions in six of seven patients, and by Zitt et al. [154] observing a 52% (complete) and 19% (partial) regression rate. Regarding the use of intravenous sodium thiosulfate in patients with calcinosis associated with autoimmune diseases, promising results were reported in a small series of patients with tumoral calcinosis associated with systemic autoimmune disorders (i.e., CREST syndrome, DM, and SLE) [155,156]. The pathogenetic mechanisms involved in sodium thiosulfate efficacy on calcinotic lesions are unknown. However, they are possibly due to calcium chelation and inhibition of vascular calcification in injured blood vessels [157]. Despite the positive clinical outcomes reported in these studies, the systemic use of sodium thiosulfate remains to be validated on a larger scale and on homogeneous patient cohorts stratified based on the underlying disease, disease activity, and comorbidities. For this reason, further randomized multicentric clinical trials should be useful to define its application in clinical practice.

Finally, adequate control of the underlying autoimmune condition could help delay the progression of dystrophic calcinosis [92].

## 8. The Potential Role of Immunosuppressive Therapies 

Over the years, several therapeutic approaches have been attempted with variable outcomes (Table 2). 

A recent systematic review by Traineau et al. [143] analyzed treatments strategies performed in patients with calcinosis associated with SSc and DM, mainly focusing on the role of some immunosuppressants such as infliximab, abatacept, rituximab, cyclophosphamide, and intravenous immunoglobulins [61,143,159,169]. 

Anti-TNF-α therapy may be considered in treating JDM and DM-associated calcinosis [75]. Adalimumab and infliximab have been shown to soften and improve calcinosis, sometimes leading to complete remission after its withdrawal [75,158]. 

In a few studies reporting contrasting results, the anti-CD20 monoclonal antibody rituximab was evaluated for treating calcinosis cutis [160,161,162,163,164,165]. Among them, studies conducted on SSc generally showed good outcomes. For example, Moazedi-Fuerst et al. [164] observed the resolution of calcinosis cutis in three patients with SSc after six months of rituximab 500 mg on day 0 and day 14, and then twice every three months, with no relapse after 1–2 years follow-up, suggesting the efficacy of B-cell depletion therapy as an option in the treatment in these patients. Similarly, other authors reported the efficacy of low-dose rituximab (four weekly infusions of 375 mg/m^2^) on calcinosis cutis in SSc patients [160,161,165,171]. On the contrary, evidence on DM/JM-related calcinosis cutis is limited and non-conclusive. A multicentric study on nine patients with severe JDM showed that calcinosis did not improve in six affected patients after rituximab administration, with mild calcinosis site infections occurring in two patients [163].

Qiblawi et al. [166] described two patients with recalcitrant calcinosis cutis who responded to apremilast, a phosphodiesterase 4 (PDE-4) inhibitor currently approved for treating psoriasis and psoriatic arthritis. The mechanism of action underlying the efficacy of this molecule in calcinosis cutis is unknown. However, the authors hypothesized that the downregulation of several inflammatory cytokines (TNF- α, interleukin (IL)-17, and IL-23) and the upregulation of the anti-inflammatory cytokine IL-10 is possibly pivotal [172]. Indeed, the elevation of proinflammatory mediators inducing chronic tissue damage, vascular hypoxia, and consequently tissue fibrosis and increased PO_4_^3−^ could become a scaffold for calcification [70]. In addition, inflammatory cytokines contribute to the formation of calcium salts in the tissue and are a driving force in the cutaneous manifestations of calcinosis cutis [70,166].

The pan-JAK inhibitor tofacitinib inhibits signaling of a wide range of type I and II cytokine receptors (i.e., IL-2, IL-4, IL-15, IL-21, IFN-γ, IL-6, IL-12, and IL-23). Tofacitinib was correlated with the improvement of calcinosis in both JDM [168] and adult refractory DM [167].

An isolated case of response to intravenous immunoglobulin (IVIG) in a woman with CREST syndrome and dystrophic calcifications was reported by Schanz et al. [170]. 

Immunosuppressive therapy should be carefully managed in case of calcinosis superinfection. Fernández-Codina et al. [66] recently reported the case of a patient with a history of DM and scleroderma overlap with positive PM/Scl-75, presenting with giant calcinosis cutis and a myopathy flare. The patient had previously developed a severely infected area of calcinosis with a subsequent septic arm during methotrexate treatment, which had been discontinued six months prior.

The common rationale for using immunosuppressive therapies in patients affected by calcinosis cutis and autoimmune diseases is reducing the inflammatory component by inhibiting different molecular targets. However, the pathogenetic pathways leading to the development of calcinosis in patients with an underlying connective tissue disease remain largely unknown. For this reason, specific targeted therapies are currently missing. Immunosuppressive therapies may be a suitable alternative in patients with refractory or recalcitrant calcinosis despite conventional therapy. However, this therapeutic approach also carries some issues, such as making the patient prone to an increased risk of infection. In addition, the clinical experience with immunosuppressive therapies in patients with calcinosis cutis is limited to case reports and case series. Moreover, there is no validated guideline for preferring one determinate immunosuppressant agent over the other or for choosing eligible patients. A better understanding of calcinosis pathogenesis together with large randomized controlled clinical trials are necessary to find novel therapeutic strategies, and provide a safe and effective target treatment for this potentially invalidating complication.

## 9. Conclusions

Calcinosis cutis and calciphylaxis are fearsome issues of autoimmune connective tissue disease. Their prevalence may vary greatly depending on the subset of the disease. Indeed, as reported above, they could be a common feature of DM and SSc, but rarely observed in SLE. However, despite calcinosis cutis and calciphylaxis being quite rare in some autoimmune conditions, due to their potentially disabling character, physicians’ awareness about the clinical presentation and management of these diseases should be increased, and their presence should be accurately evaluated in order to choose the most appropriate treatment options and avoid long-term complications. 

## Figures and Tables

**Table 1:** Prevalence, clinical features, and predictive factors for the development of calcinosis cutis associated with autoimmune connective tissue diseases.

Underlying Autoimmune Disease	Prevalence (%)	Preferential Locations	Predictive Factors for the Development of Calcinosis Cutis	References
Systemic sclerosis	18–49	hands feet	CREST a syndrome anticentromere antibodies older age decreased serum levels of fetuin-A decreased serum levels of inorganic pyrophosphate increased serum fibroblast growth factor-23 concomitance of arthritis concomitance of digital ulcers	[1,14,23,24,25,26,28]
Dermatomyositis	30–37	upper extremities lower extremities trunk	poor disease control delay in diagnosis longer disease duration dysphagia Gottron’s papules anti-MJ/NXP-2 antibodies anti- PM/Scl antibodies	[14,61]
Polymyositis	1–3	upper extremities lower extremities	longer disease duration concomitance of dermatomyositis	[14,61]
Juvenile dermatomyositis	20–70	upper extremities lower extremities trunk	early onset of disease longer disease duration poor response to conventional drugs anti-MJ/NXP-2 antibodies anti- PM/Scl antibodies abnormal nailfold capillary changes	[62,63,128,129,130]
Systemic lupus erythematosus	3–40	upper extremities buttocks peri-auricular area underneath cutaneous lupus lesions	concomitance of lupus nephritis vitamin D3 treatment poor disease control	[2,14,86,87,88]
Undifferentiated connective tissue disease	8–14	upper extremities lower extremities	Not available	[14,131]
Rheumatoid arthritis	rare	upper extremities lower extremities	Not available	[14,16,116]
Primary Sjögren’s syndrome	rare	upper extremities	Not available	[106,107,108,109,110]
Adult-onset Still’s disease	rare	hands	Not available	[126]
Juvenile polyarthritis	rare	buttocks	Not available	[127]

^a^ CREST syndrome: calcinosis, Raynaud’s phenomenon, esophageal dysmotility, sclerodactyly, and telangiectasia.

**Table 2:** Summary table of the main treatment strategies with immunosuppressant drugs used for calcinosis cutis associated with autoimmune diseases and their outcomes.

Drug	Associated Autoimmune Disease	Number of Treated Patients (N)	Dose	Outcome [Number of Patients (%)]	References
Adalimumab	DM	1	40 mg/week	CR [1 (100%)]	[75]
	JDM	28 ^a^	24 mg/m^2^ every other week ^a^	CR [8 (29%)] PR [15 (54%)]	[158]
Infliximab	DM	2	5 mg/kg/4 weeks	NR [2 (100%)]	[61]
	JDM	28 ^a^	6 mg/kg/4 weeks	CR [8 (29%)] PR [15 (54%)]	[158]
	JDM	5	3 mg/kg at weeks 0/2/6 and every 8 weeks	PR [5 (100%)]	[159]
Rituximab	CREST syndrome ^b^	1	375 mg/m^2^, 4 weekly infusions	CR [1 (100%)]	[160]
	CREST syndrome	1	375 mg/m^2^, 4 weekly infusions	CR [1 (100%)]	[161]
	DM	7	0.575–1 g/m^2^ at weeks 0/1	CR [1 (14%)] PR (NA)	[162]
	DM	2	NA	CR [0 (0%)] PR [1 (50%)]	[61]
	JDM	22	0.575–1 g/m^2^ at weeks 0/1	CR [1 (4%)] PR (NA)	[162]
	JDM	6	2 × 500 mg/m^2^ (*n* = 3) 4 × 375 mg/m^2^ (*n* = 3)	NR [6 (100%)]	[163]
	SSc	3	500 mg/m^2^ at weeks 0/2 then every 3 months	CR [3 (100%)]	[164]
	SSc	6	375 mg/m^2^ at weeks 0/1/2/3	CR [0 (0%)] PR [3 (50%)]	[165]
Apremilast	CREST syndrome Morphea	2	30 mg/day	PR [2 (100%)]	[166]
Tofacitinib	DM	3	NA	CR [3 (100%)]	[167]
	JDM	2	5–10 mgx^2^/day	CR [2 (100%)]	[168]
IVIG	ACTD	6	2 g/kg	NA [6 (100%)]	[14]
	DM	7	2 g/kg	CR [1 (14%)]	[61]
	DM	8	NA	PR [5 (63%)]	[169]
	CREST syndrome	1	2 g/kg	CR [1 (100%)]	[170]
Methotrexate	ACTD ^c^	1	20 mg/week	CR [1 (100%)]	[14]

^a^ In this study, patients were treated with infliximab alone, infliximab then adalimumab, and adalimumab alone. ^b^ CREST syndrome: calcinosis, Raynaud’s phenomenon, esophageal dysmotility, sclerodactyly, and telangiectasia. ^c^ The study included patients with dermatomyositis, systemic sclerosis, lupus panniculitis, systemic lupus erythematosus, mixed connective tissue disease, overlap connective tissue disease, undifferentiated connective tissue disease, polymyositis (*n* = 1), and rheumatoid arthritis. Abbreviations: ACTD, autoimmune connective tissue disease; CR, complete response; DM: dermatomyositis, IVIG, intravenous immunoglobulin; NA, not available; NR, no response; SSc, systemic sclerosis; PR, partial response.

## Data Availability

Not applicable.
